# Future Best Buys Can and Should Do More: Comment on "Barriers and Opportunities for WHO ‘Best Buys’ Non-Communicable Disease Policy Adoption and Implementation From a Political Economy Perspective: A Complexity Systematic Review"

**DOI:** 10.34172/ijhpm.9047

**Published:** 2025-06-09

**Authors:** Safura Abdool Karim, Marlyn Faure

**Affiliations:** ^1^Berman Institute of Bioethics, Johns Hopkins University, Baltimore, MD, USA.; ^2^Centre for the Programme of Aids Research in South Africa, University of KwaZulu-Natal, Durban, South Africa.; ^3^Ethox Centre, Oxford University, Oxford, UK.

**Keywords:** NCDs, Non-communicable Diseases, Obesity Prevention Policy, CDoH, Public Health Nutrition

## Abstract

Over a decade has passed since the World Health Organization (WHO) proposed a set of "best buys" for the prevention of non-communicable diseases (NCDs). Loffreda and colleagues’ review describes how, despite the cost-effectiveness of these interventions, their adoption is often complex and governments face many challenges in both implementing and maintaining NCD prevention policies. Industry opposition and the commercial determinants of health (CDoH) remain significant challenges to an effective NCD response. In addition, the best buys may operate inequitably and are often unable to respond to the interrelated challenges posed by the global syndemic of obesity. We suggest that the next revision of the best buys adopts a more integrated approach that prioritize structural interventions, equity-focused strategies, and mechanisms to counteract industry interference.

## Introduction

 Over a decade has passed since the World Health Organization (WHO) proposed a set of “best buys” for the prevention of non-communicable diseases (NCDs).^1^ The best buys included disease management and healthcare interventions, and, through revisions, interventions targeting NCD risk factors, such as reducing consumption of tobacco, alcohol and unhealthy foods. These include measures to tax tobacco and alcohol, reformulation such as eliminating trans-fats or reducing sodium, and restricting advertising of unhealthy commodities.^2^ Despite the best buys promise as being cost-effective interventions governments could adopt to reduce or prevent the burden of NCDs, adoption and implementation of these interventions has been mixed and are often politically fraught.^3,4^

 Loffreda and colleagues’ review^5^ reflects the complexity and ongoing challenges countries face in adopting best buys into policy. The review identifies three factors influencing the adoption of these policies which are all primarily related to government responsibilities, (though these may be influenced by non-governmental actors such as civil society organisations) namely: the ability to translate the interventions into “policy asks” or the adoption of specific laws, the ability to implement the policy and the ability to monitor and evaluate these policies – and be adaptable. These findings offer guidance for how to better advocate for and position states to adopt NCD prevention policies. Simultaneously, given the complexities of adopting the best, it is unclear whether these policy best buys remain our best “ask” of governments. Here, we must recognise that the best buys are not fixed or constant. Since 2011, the WHO has revised the best buys on a number of occasions and the most recent revision demonstrates substantial expansion from the original list of best buys (See Table). In 2023, the WHO expanded the best buys and, in doing so, added in a number of healthcare-related and medical interventions such as treating acute COPD with steroids, developing a register of patients who regularly receive prophylactic penicillin and early cancer detection and treatment.

**Table:** Best Buy Interventions to Target Risk Factors

**Risk Factor for Intervention**	**2011 Best Buy**	**2023 Best Buys**
Tobacco	1. Tax increases 2. Smoke-free indoor workplaces and public places 3. Health information and warnings 4. Bans on tobacco advertising, promotion and sponsorship	1. Increase excise taxes and prices on tobacco products 2. Implement large graphic health warnings on all tobacco packages, accompanied by plain/standardized packaging 3. Enact and enforce comprehensive bans on tobacco advertising, promotion and sponsorship 4. Eliminate exposure to second-hand tobacco smoke in all indoor workplaces, public places, public transport 5. Implement effective mass media campaigns that educate the public about the harms of smoking/tobacco use and secondhand smoke, and encourage behavior change 6. Provision of cost-covered effective population-wide support (including brief advice, national toll-free quit line services and mCessation) for tobacco cessation to all tobacco users
Alcohol	1. Tax increases 2. Restricted access to retailed alcohol 3. Bans on alcohol advertising	1. Increase excise taxes on alcoholic beverages 2. Enact and enforce bans or comprehensive restrictions on exposure to alcohol advertising (across multiple types of media) 3. Enact and enforce restrictions on the physical availability of retailed alcohol (via reduced hours of sale)
Unhealthy diet	1. Reduced salt intake in food 2. Replacement of trans fat with polyunsaturated fat 3. Public awareness through mass media on diet and physical activity*	1. Reformulation policies for healthier food and beverage products (eg, elimination of transfatty acids and/or reduction of saturated fats, free sugars and/or sodium) 2. Front-of-pack labelling as part of comprehensive nutrition labelling policies for facilitating consumers’ understanding and choice of food for healthy diets 3. Public food procurement and service policies for healthy diets (eg, to reduce the intake of free sugars, sodium, unhealthy fats, and to increase the consumption of legumes, wholegrains, fruits and vegetables) 4. Behaviour change communication and mass media campaign for healthy diets (eg, to reduce the intake of energy, free sugars, sodium, unhealthy fats, and to increase the consumption of legumes, wholegrains, fruits and vegetables) 5. Policies to protect children from the harmful impact of food marketing on diet 6. Protection, promotion and support of optimal breastfeeding practices
Physical Activity		1. Implement sustained, population wide, best practice communication campaigns to promote physical activity, with links to community-based programmes and environmental improvements to enable and support behaviour change

* Diet and physical activity were combined in the 2011 best buys but separated in later versions of the best buys. Source: Authors construction based on the 2011^1^ and 2023^6^ best buys.

 In addition to this, the 2023 revision also saw the expansion of and an increased specificity to interventions targeting unhealthy commodities – most notably in relation to unhealthy food where the best buys have incrementally expanded. These now include measures such as front of package labelling,^7^ restrictions on child-directed marketing^8^ and reformulation policies.^9^ It is thus possible for the best buys to be further strengthened, expanded or amended to better support NCD prevention efforts. Our commentary reflects on the value of the best buys in light of Loffreda and colleagues’ findings about the difficulties in adopting them. Additionally, we draw attention to the need to consider equity in formulating and identifying as an important issue that has been somewhat overlooked, and is not expressly dealt with in Loffreda and colleagues’ review.

## The Best Buys Provide an Important Lodestar for NCD Prevention Efforts

 The best buys offer a set of public health interventions that are likely to be effective, yet cost-effective, which is of particular importance for low- and middle-income countries (LMICs).^1^ In addition, many of the interventions can be translated into clear, concrete legal and policy interventions and thus more easily implemented though Loffreda et al note, there is a need to tailor the recommendations to particular contexts.^10^ In addition, many of the best buys can be “win-wins” for government, such as excise taxes on unhealthy commodities that both reduce consumption, and generate income for governments.^3^

 The best buys also offer normative guidance to countries about what kinds of interventions to adopt to prevent NCDs.^11^ The endorsement and adoption of best buys through the World Health Assembly further buttresses the best buys’ status as a goal for countries to pursue. Inclusion as a best buy provides a basis for public health advocates to argue for that intervention’s adoption, and, through evaluations such as the Noncommunicable Diseases Progress Monitor,^12^ a rubric against which governments’ progress on NCD prevention can be measured. Moreover, given the evidence-based of best-buys, it provides states, especially in LMICs, with support against opposition and legal challenges.^13^

## Yet, There Are Many Challenges to the Adoption of Best Buys

 First, policies which target unhealthy commodities often implicate the commercial determinants of health (CDoH)and harm the commercial interests of the producers of these unhealthy commodities. The industry opposition to these policies is often well-orchestrated, coordinated and heavily funded, making adoption challenging. In addition, industry will often position these policies, and their public health objectives, as being in tension with economic, casting governments as having to choose between prioritising economic development or the health of their population.^14,15^ It is also difficult for smaller LMIC governments to address the highly funded efforts of opposition from multi-national corporations.^16^ Critically, in most countries the challenges faced in adopting these policies do not end once the policies are in place.

 Often, opposition to these policies is unrelenting. When policies are eventually adopted, they may be subject to legal challenges in the courts or, as was observed in the case of South Africa’s sugary beverage tax, continued lobbying and opposition through media campaigns from sugar and sugary beverage industry organizations resulting in a moratorium on the tax and a pervasive narrative that the tax has killed the sugar industry.^17,18^ Public health advocates and governments are unlikely to have the resources and political will to continue to fight for these policies indefinitely. This is not to say that government should not pursue these policies but to acknowledge that there are continuing challenges to maintain these policies.

 Second, in addition to the implantation challenges noted by Loffreda et al, we argue that the best buys represent small policy wins in an effort to combat the NCD epidemic, rather than integrated measures to support radical changes to or transformation of the systems that drive the NCD epidemic. While best buys are important, in our view, they are not intended to be silver bullets to a complex NCD epidemic nor are they, necessarily, the most effective interventions a country could adopt. The best buys can lead to changes and modifications in the existing food system but they address only a narrow band of unhealthy foods, primarily targeting unhealthy ingredients, rather than aiming for food systems transformation. Moreover, as Loffreda et al note, this means that governments often perceive a tension in addressing unhealthy foods, or unhealthy commodities in contexts with other pressing issues that emanate from fragility such as undernutrition, poverty and high burdens of communicable diseases.

 There has also been a recognition of the intersections of these challenges. The *Lancet* Commission on obesity describes these intersecting challenges of obesity, communicable diseases and climate change as a global syndemic that requires coordinated action through interventions that address more than one challenge simultaneously.^19^ Given these inter-related challenges with inter-related structural drivers, it is worth identifying and creating integrated approaches that address these challenges together – such as double- and triple-duty interventions and addressing the structural drivers of these epidemics, including CDoH. Doing this requires a more ambitious and holistic set of interventions than the best buys which, in their present construction, very narrowly focus on NCDs. There is no reason to limit the best buys to single-duty interventions and future revisions of the best buys could begin to more explicitly consider an integrated approach to addressing these intersecting health challenges.

 Third, there is a need to recognise that restrictive policies often implicate equity and not always in a positive way. The NCD burden is not spread equitably, it burdens the poor, vulnerable and creates the vulnerability that Loffreda et al highlight in their findings without explicitly linking this to issues of equity. While the poor benefit the most from restrictive policies, they also bear greater burdens under these policies.^20^ Governments and public health advocates should consider complex equity measures, and how they intersect, to include socioeconomic, gender race and other markers, to including how various groups are differentially impacted in different geographic settings. This may involve expanding the currently restrictive best buy interventions, to include or be paired with measures that provide benefits to poor and vulnerable groups. For example, we should not only support taxation of unhealthy foods but also food subsidies. Our goal should not just be to reduce consumption of unhealthy products but to make easier the choice to live a healthy life. A more equitable set of best buys would aim to identify cost-effective interventions that do not unduly unburden vulnerable populations.

## Rethinking the Best Buys: A More Integrated Approach

 The WHO Best Buys have played a crucial role in advancing global NCD prevention efforts, but their normative power should be leveraged upon to adopt a more integrated approach to addressing the broader determinants of health more effectively.

 Academic research, robust evidence and an active civil society can all be important in supporting the adoption of policies to improve health but these too have their challenges. This may also move beyond hegemonic multilateralism, towards solidarity between states, to foster transformed economic, political and social systems to center population and planetary health as primary aims. Efforts to address CDoH may benefit from drawing on these more radical and structural approaches.

 NCD prevention remains complex and contested, requiring sustained advocacy and integrated policy approaches. Future iterations of the best buys should prioritize structural interventions, equity-focused strategies, and mechanisms to counteract industry interference. Only through a more ambitious and coordinated effort can we achieve meaningful progress in reducing the global NCD burden.

## Ethical issues

 Not applicable.

## Conflicts of interest

 Authors declare that they have no conflicts of interest.
